# Hypothermia for perinatal asphyxia: trial-based resource use and costs at 6–7 years

**DOI:** 10.1136/archdischild-2017-314685

**Published:** 2018-07-11

**Authors:** Oliver Rivero-Arias, Oya Eddama, Denis Azzopardi, A David Edwards, Brenda Strohm, Helen Campbell

**Affiliations:** 1 Nuffield Department of Population Health, National Perinatal Epidemiology Unit (NPEU), University of Oxford, Oxford, UK; 2 Centre for the Developing Brain, King’s College London, London, UK; 3 Institute of Clinical Sciences, Imperial College, London, UK

**Keywords:** perinatal asphyxial encephalopathy, moderate hypothermia, cost, long-term, disability

## Abstract

**Objective:**

To assess the impact of hypothermic neural rescue for perinatal asphyxia at birth on healthcare costs of survivors aged 6–7 years, and to quantify the relationship between costs and overall disability levels.

**Design:**

6–7 years follow-up of surviving children from the Total Body Hypothermia for Neonatal Encephalopathy (TOBY) trial.

**Setting:**

Community study including a single parental questionnaire to collect information on children’s healthcare resource use.

**Patients:**

130 UK children (63 in the control group, 67 in the hypothermia group) whose parents consented and returned the questionnaire.

**Interventions:**

Intensive care with cooling of the body to 33.5°C for 72 hours or intensive care alone.

**Main outcome measures:**

Healthcare resource usage and costs over the preceding 6 months.

**Results:**

At 6–7 years, mean (SE) healthcare costs per child were £1543 (£361) in the hypothermia group and £2549 (£812) in the control group, giving a saving of −£1005 (95% CI −£2734 to £724). Greater levels of overall disability were associated with progressively higher costs, and more parents in the hypothermia group were employed (64% vs 47%). Results were sensitive to outlying observations.

**Conclusions:**

Cost results although not significant favoured moderate hypothermia and so complement the clinical results of the TOBY Children study. Estimates were however sensitive to the care requirements of two seriously ill children in the control group. A quantification of the relationship between costs and levels of disability experienced will be useful to healthcare professionals, policy makers and health economists contemplating the long-term economic consequences of perinatal asphyxia and hypothermic neural rescue.

**Trial registration number:**

This study reports on the follow-up of the TOBY clinical trial: ClinicalTrials. gov number NCT01092637.

**What is already known on this topic?**

- The benefits of hypothermic neural rescue in terms of cognitive and disability-free survival persist into middle childhood.
- Data on long-term costs following perinatal asphyxia and hypothermia have not previously been reported.

**What this study adds?**

- New comparative data on healthcare costs at 6–7 years after hypothermia plus intensive care and intensive care alone for perinatal asphyxia.
- A quantification of healthcare costs by level of disability, in survivors of perinatal asphyxia.

## Introduction

A deficiency of oxygen at birth can result in injury to the neonatal brain. This neurological syndrome known as perinatal asphyxial encephalopathy can be characterised by the need for resuscitation, neurological depression and seizures, and is associated with a high risk of death or early neurodevelopmental impairment.^1^ Initiating moderate hypothermia soon after delivery for 72 hours has been shown to reduce the risk of death or disability at 18–24 months of age, and to increase the rate of survival without disability.^2^ Long-term follow-up of children in three major trials of hypothermia (the Total Body Hypothermia for Neonatal Encephalopathy (TOBY) trial, the CoolCap trial and the National Institute of Child Health and Human Development (NICHD) trial) has confirmed that the clinical benefit observed at 18 months persists or is maintained, at least in part, into middle childhood.^3–5^

In addition to clinical data, the generation of data on the long-term cost impact of perinatal asphyxial encephalopathy and hypothermia is also crucial. Such data can provide clinicians and policy makers with vital information about the ongoing resources needed to provide care for survivors with disabilities of varying severities. Long-term cost data are also necessary inputs into cost-effectiveness analyses, which are paramount to ensure that scarce healthcare resources are appropriately invested in technologies that will realise most patient benefit. Indeed, cost-effectiveness analysis is now an integral part of the health technology appraisal process in many countries.^6–8^

The short-term cost-effectiveness of hypothermia for perinatal asphyxial encephalopathy has been modelled by synthesising outcome data from the TOBY, CoolCap and NICHD trials and using data on healthcare costs to 18 months from TOBY.^9^ Acknowledging the long-term implications of the condition (which can include cerebral palsy, functional disability and cognitive impairment), the authors presented a sensitivity analysis in which the time horizon for the analysis was extended from 18 months to 18 years. In the absence of long-term data from neonatal encephalopathy survivors however, data on costs were taken from a cohort study of preterm infants and this was acknowledged as a limitation of the analysis.^10 11^

To address this lack of long-term data and as part of the 6–7 years follow-up of surviving children in the TOBY trial, data were collected on children’s healthcare resource use and health-related quality of life (HRQoL).^5 12^ The objectives of this substudy were to describe resource use and costs at 6–7 years in each trial arm and to quantify healthcare costs by overall disability level.

## Methods

### Study population

Parents of surviving children who participated in the original TOBY trial were invited to take part in the follow-up TOBY Children study when their child reached 6–7 years.^5^ Detailed clinical findings from TOBY Children, which captured outcomes for 280 of the 325 infants recruited to the original TOBY trial, are reported elsewhere.^5^ In brief, they showed at 6–7 years, when compared with the control group, a higher number of children in the hypothermia group survived with an IQ score ≥85 (52% vs 39%, relative risk (RR) 1.31, p=0.04), survived without neurological abnormalities (45% vs 28%, RR 1.60; 95% CI 1.15 to 2.22), and had significant reductions in the risk of cerebral palsy (21% vs 36%, p=0.03) and of moderate or severe disability (22% vs 37%, p=0.03).^5^

As part of that study, parents received a postal questionnaire including questions about the use of healthcare services by their child over the previous 6 months and the HRQoL Health Utilities Index (results of which are reported elsewhere).^13^ The TOBY trial included centres from the UK, Sweden, Hungary, Finland and Israel, with UK centres contributing over 85% to the total sample size.

### Healthcare resource use and costs

The questionnaire contained a list of healthcare professionals and services likely to be used by TOBY Study children across primary, community and secondary care (column 1 of table 1). Parents recorded whether their child had any contact with each professional/service in the previous 6 months as well as the number of contacts. For contacts with hospitals, parents reported the reason, and the duration and ward type for any inpatient admissions. Parents could also report contacts with any other professionals (shown in column 1 of table 1 under the heading of ’Miscellaneous clinics/therapist sessions').

**Table 1:** Healthcare contacts included in the cost analysis and their associated unit costs (expressed in 2015/2016 UK pounds)

Resource use item	Unit cost (£)	Source
Primary care:		
GP clinic consultation	36	Per typical 9.22 min consultation. Section 10.3b, Unit Costs of Health and Social Care 2016.^14^
Practice nurse consultation	11	Per typical 15.5 min consultation. Section 10.2, Unit Costs of Health and Social Care 2016.^14^
Community care:		
Health visitor contact visit	59	Weighted average of Community Health Services Health Visitor and Midwifery (HVM) codes N03F and N03G. NHS Reference Costs 2015/2016.^15^
Community nurse contact visit	112	Weighted average of Community Health Services Nursing (NURS) codes N06CF, N12 and N29CF. NHS Reference Costs 2015/2016.^15^
Community paediatrics contact visit	278	Community Paediatrics Outpatient Attendance (Code 290). NHS Reference Costs 2015/2016.^15^
Optician contact visit	21	http://www.fodo.com/downloads/ofnc-sight-test-fee-statement---final.pdf 35
Orthoptist contact visit	58	Orthoptics Outpatient Attendance (Code 655). NHS Reference Costs 2015/2016.^15^
Physiotherapist contact visit	87	Physiotherapist, Child, One to One Contact (Code A08C1). NHS Reference Costs 2015/2016.^15^
Speech and language therapist contact visit	94	Speech and Language Therapist, Child, One to One Contact (Code A13C1). NHS Reference Costs 2015/2016.^15^
Secondary (hospital-based) care:		
A&E visit	187	Weighted average of all Emergency Medicine contact codes excluding cases DOA. NHS Reference Costs 2015/2016.^15^
Hospital day unit attendance	749	Weighted average of Day Case Paediatric Admissions for Unexplained Symptoms in children with comorbid condition score 0 (code PX56B) and comorbid condition score 1+ (PX56A). NHS Reference Costs 2015/2016.^15^
Hospital outpatient attendance	194	Paediatrics Outpatient Attendance (Code 420). NHS Reference Costs 2015/2016.^15^
Hospital inpatient bed day	431	Weighted average of non-elective Excess Bed Day Costs across all paediatric codes. NHS Reference Costs 2015/2016.^15^
Intensive care inpatient bed day	1173	Basic Paediatric Critical Care, code XB07Z. NHS Reference Costs 2015/2016.^15^
Miscellaneous clinics/therapist sessions:		
Occupational therapist contact visit	131	Occupational Therapist, Child, One to One Contact (Code A06C1). NHS Reference Costs 2015/2016.^15^
Parent support worker/special educational needs worker contact visit	52	Per hour of client-related contact for a Family Support Worker. Section 11.8. Unit Costs of Health and Social Care 2016.^14^
Dietician contact visit	81	Community Health Services, Dietician Contact (Code A03). NHS Reference Costs 2015/2016.^15^
Dentist contact visit	54	Per contact for a mid-band treatment (band 2). NHS Dental Charges. Section 10.7. Unit Costs of Health and Social Care 2016.^14^
Educational psychologist contact visit	85	Per hour of client-related contact. http://www3.hants.gov.uk/servicesforschools/education-psychology/education-psychology-prices.htm. 36
Home Respite Team contact visit	24	Per family per hour for Home Support. Section 6.12 Short-break provision for disabled children and their families. Unit Costs of Health and Social Care 2016.^14^
Social worker contact visit	79	Per hour of client-related contact for a Social Worker (Children’s Services). Section 11.3. Unit Costs of Health and Social Care 2016.^14^
Support for physical disability contact visit	24	Per family per hour for Home Support. Section 6.12 Short-break provision for disabled children and their families. Unit Costs of Health and Social Care 2016.^14^

A&E, accident and emergency; DOA, dead on arrival; GP, general practitioner.

For each child, costs were estimated by multiplying the numbers and/or durations recorded for each type of contact by appropriate unit costs obtained primarily from national sources.^14 15^ These unit costs are also shown in table 1, expressed in 2015–2016 UK Pound Sterling.

### Statistical analysis

It is accepted that clinical practice, healthcare resource use and unit costs can vary between countries, and so simply aggregating multinational data for a cost analysis will likely generate findings that are not meaningful or representative to any one country.^16 17^ In view of this and with 89.7% of questionnaire responses received from the UK (9.7% were from Hungary, and 0.6% from Finland), only UK data were used for the analysis.

Mean (SE) healthcare contact and cost data were summarised for each trial arm and compared using mean differences and 95% CIs. Data were right skewed but as parametric and non-parametric CIs were similar, only parametric intervals are reported.^18^

Data were missing for 11.5% of healthcare contacts and were assumed to be missing at random. Two types of missingness were identified: one, whereby the type of contact was recorded but the number of contacts was missing, the other where data on both the type and number of contacts were missing. Multiple imputation (MI) using chained equations was used to impute missing values (see online supplementary appendix).^19 20^ The base-case cost results were generated using the MI data, with Rubin’s rule used to generate combined estimates of means and SEs across MI datasets.^21^

10.1136/archdischild-2017-314685.supp1 Supplementary file 1

In addition to the comparative cost analysis, the relationship between total healthcare costs and overall disability levels at 6–7 years was explored. Given the skewness in the data, a generalised gamma model with a log link function was used to regress total costs against overall disability levels (none, mild, moderate and severe—see footnote to table 2 for level descriptions), while controlling for trial arm and characteristics at trial entry (delivery complications at birth (yes/no), gestational age at birth (weeks), birth weight (g) and gender). A modified Park test was used to confirm the appropriateness of the gamma model.^22 23^ Rubin’s rule was also implemented to generate the combined regression coefficients and associated SEs across MI datasets.

**Table 2:** Child demographics, clinical characteristics and neurological function at 6–7 years by trial arm, for surviving children in the UK with questionnaire data. Also shown are parental socioeconomic characteristics

	Control group (n=63)	Hypothermia group (n=67)	P values
Baseline demographics and characteristics at trial entry			
Male sex, n (%):	34 (54)	42 (63)	0.31
Missing	0	0	
Age (years)			
Median (IQR)	6.2 (6.1–6.5)	6.3 (6.1–6.9)	0.37
Missing	3	1	
Gestational age (weeks):			
Median (IQR)	40 (39-41)	40.3 (39.3–41.4)	0.33
Missing	4	0	
Birth weight (g):			
Median (IQR)	3400 (3194–3930)	3450 (3175–3838)	0.81
Missing	0	0	
Delivery complications, n (%):	46 (74)	51 (76)	0.80
Missing	1	0	
Apgar score≤5 at 10 min, n (%):	38 (72)	39 (72)	0.95
Missing	10	13	
Outcomes at 6–7 years			
Normal neurological function, n (%)	31 (49)	46 (69)	0.02
Missing	0	0	
IQ≥85, n (%)	40 (67)	54 (81)	0.07
Missing	3	0	
Overall disability*, n (%):			
None	27 (43)	49 (75)	<0.001
Mild	10 (16)	4 (6)	
Moderate	8 (13)	4 (6)	
Severe	18 (29)	8 (12)	
Missing	0	2	
Parental socioeconomic characteristics at 6–7 years			
Main carer highest qualification, n (%):			0.81
None of the below	2 (3)	5 (7)	
Vocational qualification NVQ or CSE	9 (15)	9 (13)	
O Level, GCSE or Scottish Standards	14 (23)	11 (16)	
BTEC, A Levels or Scottish Highers	6 (10)	6 (9)	
Diploma or HND	6 (10)	11 (16)	
University degree	12 (20)	15 (22)	
Postgraduate university degree	10 (16)	9 (13)	
Other qualification	2 (3)	1 (1)	
Missing	2	0	
Main carer employment, n (%):			0.02
Employed	22 (36)	41 (61)	
Self-employed	7 (11)	2 (3)	
Unemployed	14 (23)	10 (15)	
Other†	18 (30)	14 (21)	
Missing	2	0	
Main carer home, n (%):			0.89
Owner (mortgage)	40 (63)	43 (64)	
Council rented	11 (17)	7 (10)	
Private rented (furnished)	3 (5)	2 (3)	
Private rented (unfurnished)	3 (5)	11 (16)	
Housing society or co-operative	3 (5)	1 (1)	
Other‡	3 (5)	3 (4)	
Missing	0	0	

*Overall disability—mild disability (an IQ score of 70–84, level 1 gross motor function (is able to walk independently but may have some gait abnormalities), or abnormality in one or both eyes with normal or nearly normal vision); moderate disability—(an IQ score of 55–69, level 2 or 3 gross motor function (has minimal ability to perform gross motor skills or requires assistance with walking), or moderately reduced vision); severe disability (an IQ score of <55, level 4 or 5 gross motor function (needs adaptive seating or has severely limited mobility), or no useful vision).

†Open-ended question (responses included housewife, carer, etc).

‡Open-ended question.

A Level, advanced level; BTEC, Business and Technology Education Council; CSE, Certificate of Secondary Education; HND, Higher National Diploma; O Level, ordinary level; GCSE, General Certificate of Secondary Education; IQR, Inter-quartile range; NVQ, National Vocational Qualification.

All analyses were conducted in Stata MP V.13 (Stata Statistical Software, 2013, StataCorp, College Station, Texas, USA).

### Sensitivity analysis

Sensitivity analysis explored the significance of outlying observations; two children in the control arm spent considerably more nights in hospital than other children requiring inpatient care. In a first analysis, the two children were removed and in a second analysis, their days in hospital were replaced by the longest length of stay observed among other children requiring inpatient care (20 days).

## Results

### Study population

**Figure 1:**
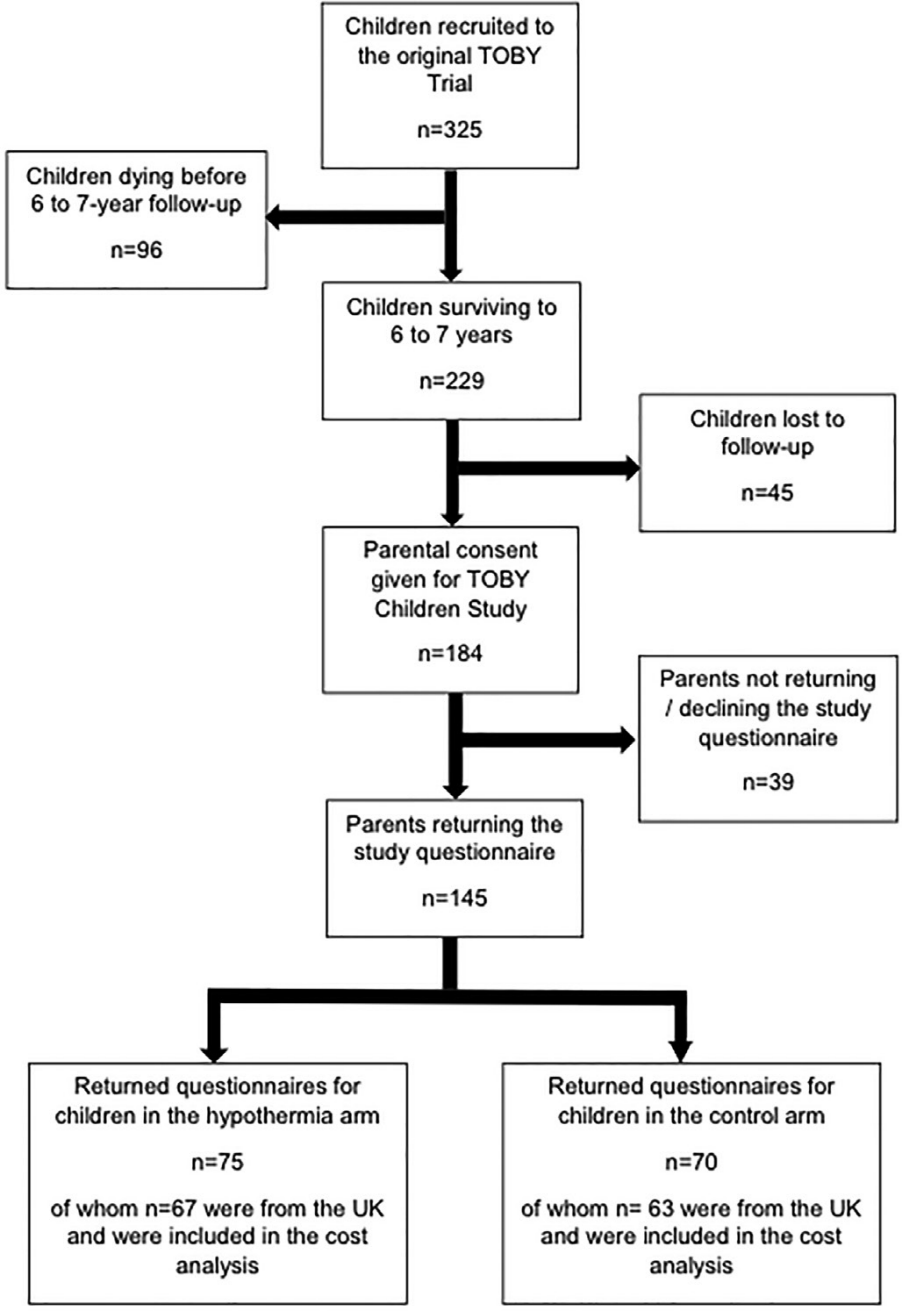
Flow of children through the study. TOBY, Total Body Hypothermia for Neonatal Encephalopathy.

Figure 1 shows the flow of children through the study. Of the 229 TOBY trial survivors at 6–7 years, 184 (80%) parents/carers consented to participate in the main TOBY Children study and 45 (20%) were lost to follow-up. One hundred and forty-five of the 184 consenting parents (79%) returned the study questionnaire and 39/184 (21%) either did not respond or declined the questionnaire. The 130/145 (90%) responses from the UK form the sample for this study.

Sixty-seven children were from the hypothermia arm and 63 from the control arm. There were no differences between arms with regard to baseline demographics or clinical characteristics at trial entry (table 2). Differences in clinical outcomes at 6–7 years were comparable to those observed for survivors in the wider TOBY Children study population; a higher proportion of children in the hypothermia group had normal neurological functioning and an IQ score ≥ 85, and fewer had moderate or severe levels of disability. More carers were in employment in the hypothermia group.

There were no differences between the 145 children whose parents returned the study questionnaire and the subsample of 130 UK children (online supplementary appendix table A1). There were also no differences between the 130 UK children and the 39 children whose parents consented to the study but did not return the questionnaire (online supplementary appendix table A1). Among these 39 non-responding families (and as observed for the responding families), a higher proportion of children in the hypothermia group had an IQ≥85 (16/23 (70%) vs 9/16 (56%), p=0.394) and had normal neurological functioning (14/23 (61%) vs 6/16 (38%), p=0.151).

Of the 45/229 (20%) surviving children who were lost to follow-up, 18 (40%) were in the hypothermia arm and 27 (60%) were in the control arm.^5^ When compared with surviving children for whom primary outcome data were available, those lost to follow-up had a higher frequency (although not statistically significantly so) of severe abnormalities on amplitude-integrated electroencephalogram (EEG) at trial entry, and lower scores on the Mental Development Index at 18 months.^5^

### Health service resource use and associated costs

Base-case mean healthcare contacts and costs estimated using the multiple imputation data are summarised in table 3 (additional details of the missing data, and the impact of multiple imputation are discussed in the online supplementary appendix, tables A2–A4).

**Table 3:** Base-case mean (SE) healthcare resource use and cost (UK £ 2015/2016) by trial arm, and mean differences (95% CI) between trial arms (UK data only, n=130)*

Resource use category	Control group (n=63) Mean (SE)	Hypothermia group (n=67) Mean (SE)	Mean difference (95% CI)	Control group (n=63) Mean cost (SE)	Hypothermia group (n=67) Mean cost (SE)	Mean cost difference (95% CI)
Primary care						
GP clinic visits	1.46 (0.24)	1.37 (0.26)	−0.09 (–0.79 to 0.60)	£52 (£8)	£49 (£9)	–£3 (–£28 to £22)
Practice nurse visits	0.12 (0.04)	0.35 (0.13)	0.23 (–0.05 to 0.51)	£1 (£0)	£4 (£1)	£3 (–£1 to £6)
Total cost—primary care	–	–	–	**£54 (£9)**	**£53 (£10)**	**–£1 (–£27 to £26)**
Community care						
Health visitor visits	0.06 (0.06)	0.18 (0.08)	0.12 (–0.09 to 0.33)	£3 (£4)	£11 (£5)	£7 (–£5 to £20)
Community nurse visits	0.80 (0.33)	0.85 (0.50)	0.05 (–1.16 to 1.26)	£89 (£37)	£95 (£56)	£6 (–£130 to £141)
Community paediatrics visits	0.29 (0.09)	0.57 (0.21)	0.28 (–0.19 to 0.75)	£81 (£26)	£159 (£59)	£78 (–£52 to £208)
Optician visits	0.27 (0.07)	0.45 (0.13)	0.17 (–0.12 to 0.47)	£6 (£1)	£10 (£3)	£4 (−£3 to £10)
Orthoptist visits	0.30 (0.10)	0.25 (0.12)	−0.06 (–0.36 to 0.25)	£17 (£6)	£14 (£7)	–£3 (–£21 to £14)
Physiotherapist visits	3.07 (0.89)	2.46 (1.11)	−0.61 (–3.54 to 2.33)	£268 (£78)	£215 (£97)	–£53 (–£309 to £203)
SALT visits	2.64 (0.71)	3.03 (1.47)	0.39 (–2.95 to 3.74)	£249 (£67)	£287 (£139)	£37 (–£279 to £353)
Total cost—community care	–	–	–	**£715 (£159)**	**£790 (£235)**	**£75 (–£504 to £655)**
Secondary (hospital-based) care:						
A&E visits	0.42 (0.16)	0.19 (0.06)	−0.23 (–0.55 to 0.09)	£78 (£29)	£35 (£11)	–£43 (–£103 to £17)
Hospital day unit visits	0.10 (0.07)	0.14 (0.07)	0.04 (–0.15 to 0.22)	£75 (£51)	£101 (£49)	£27 (–£114 to £167)
Hospital outpatient visits	0.87 (0.26)	0.87 (0.22)	−0.01 (–0.67 to 0.65)	£170 (£50)	£169 (£44)	–£1 (–£129 to £127)
Hospital inpatient days	2.21 (1.25)	0.69 (0.38)	−1.51 (–4.04 to 1.01)	£1116 (£668)	£299 (£163)	–£817 (–£2141 to £508)
Total cost—secondary care	–	–	–	**£1438 (£732)**	**£604 (£199)**	**–£834 (–£2295 to £627)**
Other miscellaneous clinics/therapist sessions†:						
Occupational therapist visits	1.14 (0.55)	0.50 (0.24)	−0.64 (–1.81 to 0.53)	£149 (£72)	£65 (£32)	–£84 (–£237 to £69)
Parent support worker/special educational needs worker	1.02 (0.82)	0.24 (0.14)	−0.78 (–2.38 to 0.83)	£53 (£42)	£12 (£7)	–£40 (–£124 to £43)
Dietician visits	0.05 (0.05)	0.10 (0.07)	0.06 (–0.11 to 0.22)	£4 (£4)	£8 (£6)	£5 (–£9 to £18)
Dentist visits	0.02 (0.02)	0.04 (0.03)	0.03 (–0.03 to 0.09)	£1 (£1)	£2 (£1)	£2 (–£2 to £5)
Educational psychologist visits	0.02 (0.02)	0.01 (0.01)	−0.00 (–0.04 to 0.04)	£1 (£1)	£1 (£1)	£0 (–£4 to £4)
Home respite team visits	0.38 (0.38)	0.00 (0.00)	−0.38 (–1.11 to 0.35)	£73 (£73)	£0 (£0)	−£73 (−£213 to £67)
Social worker visits	0.00 (0.00)	0.09 (0.09)	0.09 (–0.09 to 0.27)	£0 (£0)	£7 (£7)	£7 (–£7 to £22)
Support for physical disability visits	0.32 (0.32)	0.00 (0.00)	−0.32 (–0.93 to 0.29)	£61 (£61)	£0 (£0)	–£61 (–£178 to £56)
Total cost—miscellaneous clinics/therapist sessions	–	–	–	**£342 (£157)**	**£97 (£35)**	**–£245 (–£555 to £64)**
Total overall cost	–	–	–	**£2549 (£812)**	**£1543 (£361)**	**–£1005 (–£2734 to £724)**

The bold values identify the total costs for each cost category (eg, primary care, community care etc) and for overall total costs.

*Base-case results estimated using imputed data.

†Other miscellaneous clinics/therapist sessions are from a section of the questionnaire which was optional to complete. If respondents did not complete this section, we have assumed no contacts took place.

A&E, accident and emergency; GP, general practitioner; SALT, speech and language therapy.

For many of the healthcare contacts, mean usage and costs were similar in both trial arms (table 3). In contrast, inpatient admission costs were noticeably lower in the hypothermia group (mean difference −£817 (−£2141 to £508)). Although the proportion of children with at least one hospital inpatient admission in each arm was similar (11.1% vs 10.8%), in the control arm the mean total time in hospital was greater (19.9 vs 6.6 days) due to two children spending a total of 53 and 58 days in hospital (see online supplementary appendix table A4). One admission for epilepsy required a prolonged stay (14 days) in intensive care.

Children in the control group also appeared to need greater input from other specialists. The mean cost difference for these ‘other’ services favoured the hypothermia group at −£245 (−£555 to £64), but was not statistically significant.

The total mean NHS cost per child over the 6-month period was £2549 (£812) in the control group and £1543 (£361) in the hypothermia group, giving a non-statistically significant difference of −£1005 (−£2734 to £724) favouring hypothermia.

### Costs and disability levels

When modelling costs, the modified Park test showed the gamma model to be most appropriate when compared with models using Poisson, inverse Gaussian and Gaussian distributions. Table 4 shows the estimated coefficients from the regression model. The multiplicative effect of each coefficient is also reported. The coefficients for each disability level increase with severity, and those for moderate and severe levels are statistically significantly greater than the reference category of no disability. The model was used to estimate mean total healthcare costs for each of the four disability levels for an average child in the TOBY trial (a male, born at 40 weeks, having delivery complications, a birth weight of 3400 g and receiving hypothermia) (see online supplementary appendix for the approach used). The resulting estimated mean total cost for a child with no disabilities was £450, with mild disabilities was £1043 (95% CI £363 to £2998), with moderate disabilities was £5651 (95% CI £1715 to £18 624) and with severe disabilities was £12 335 (95% CI £5238 to £29 049).

**Table 4:** Generalised linear regression equation (gamma model with a log link) of the relationship between total healthcare costs and overall disability levels (n=125 UK children)*

Variable	Coefficient	SE	P values	Multiplicative effect
Trial arm†	0.552	0.374	0.140	1.74
Delivery complications‡	0.597	0.390	0.126	1.82
Gestational age	−0.145	0.121	0.230	0.87
Birth weight	−0.0002	0.0003	0.393	1.00
Male gender§	−0.286	0.336	0.395	0.75
Mild disability¶	0.841	0.538	0.119	2.32
Moderate disability¶	2.531	0.60	0.000	12.56
Severe disability¶	3.311	0.437	0.000	27.43
Constant	11.850	4.471	0.008	–

*Five children were missing characteristics at trial entry and were excluded from the model.

†Hypothermia arm relative to base category of control arm.

‡Relative to base category of no delivery complications.

§Relative to base category of female.

¶Relative to base category of no disability.

### Sensitivity analysis

**Figure 2:**
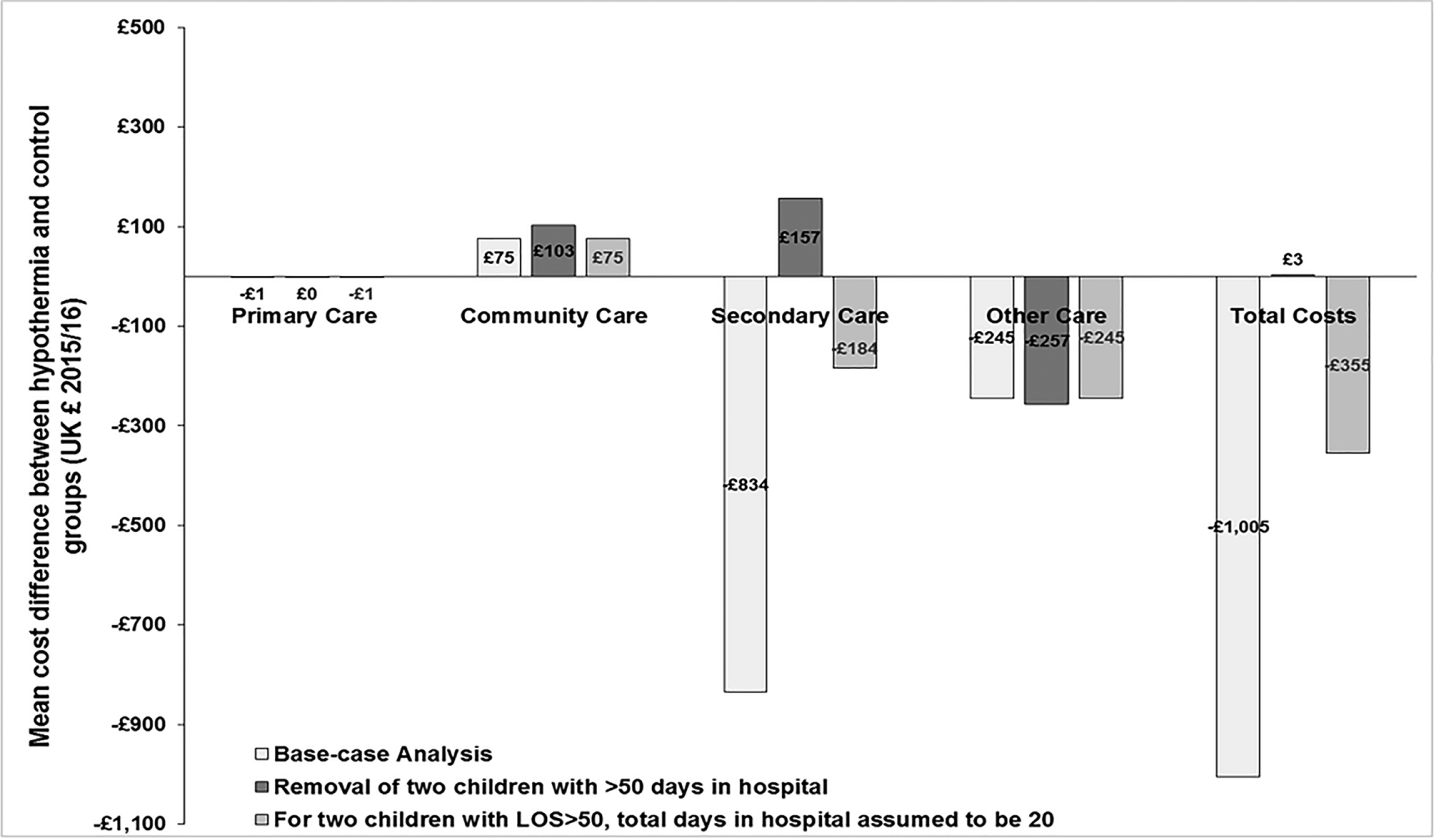
Mean cost differences between hypothermia and control groups for different healthcare sectors and overall. Results shown for the base-case and sensitivity analysis. LOS, length of stay.

Excluding the two children in the control arm who each spent >50 days in hospital had a substantial impact on the comparative cost results. Figure 2 shows how the mean total cost difference of −£1005 favouring hypothermia disappeared and the mean cost saving in secondary care of −£834 was replaced by a cost increase of £157. Retaining both children but replacing their time in hospital with 20 days reduced the mean secondary care cost difference from −£834 to −£184 and the mean total cost difference by two-thirds from −£1005 to −£355 in favour of hypothermia.

Re-estimating the regression model after removing the two outlying children reduced the coefficient for the severe disability level from 3.31 to 2.68, that is, relative to a child with no disability, costs for a child with a severe disability were estimated to be 14 times greater rather than 27 times greater as in the base-case analysis (table 4).

## Discussion

This study is the first to offer a comparative assessment of primary, community and secondary sector healthcare costs in children aged 6–7 years after randomisation to standard care with hypothermia or standard care alone, for perinatal asphyxia. On average, those randomised to hypothermia had lower although non-statistically significant total healthcare costs during the previous 6 months. As the follow-up of a randomised trial and using a sample size fixed from the original trial, we were underpowered to detect statistically significant differences in costs. This is not unusual for clinical trials which are routinely powered on clinical outcomes. When considered alongside the main clinical outcomes from the TOBY Children study, the direction of the cost figures in favour of hypothermia appears intuitive.^5^

Results were highly sensitive to the hospital inpatient length of stays of two children in the control group. Both had severe neurodevelopmental disability and multiple handicaps and spent considerably more days in hospital than any other children in the study. During the 6-month recall period, total healthcare costs for these two children amounted to £26 477 and £40 129 and demonstrate that the needs of children with multiple severe sequelae can be substantial.

Whether the magnitude of the cost differential between trial arms is representative, will depend on whether the care requirements for these two children are typical. During the 6-month study period, we may have captured unusually severe manifestations of their chronic conditions, which if used as the basis of long-term cost extrapolations, could lead to erroneous predictions. For policy makers and health economists, the 6-month time horizon used in this study is a limiting factor and the uncertainties arising as a consequence of this should be borne in mind.

Few comparable estimates of long-term resource use for this patient group have been reported. The NICHD follow-up study reported comparative data on the proportion of children receiving speech therapy at 6–7 years, but not on the number of contacts that took place.^24^ The proportions for the hypothermia and control groups of 30% and 43%, respectively were not dissimilar to those observed for speech and language therapy in this study; 25% (15/59) in the hypothermia arm and 48% (28/58) in the control arm.

We followed convention for modelling costs and used a generalised gamma model with a log link function when exploring the relationship between healthcare costs and disability levels.^25^ Relative to children with no disability, progressively higher costs were incurred by those with mild, moderate and severe disabilities. Findings were again sensitive to the two outlying children; in their absence, costs for a child with a severe disability were estimated to be 14 times greater than for a child with no disability, rather than 27 times greater as in the base-case analysis.

Our analysis has a number of limitations. First, the perspective for the costing work was restricted to the health service and excluded costs borne by the family and wider society. Table 2 showed that fewer parents of children in the control group were in paid employment, probably because of the demands of caring for an unwell child.^26^ This likely translates into a reduction of family income and an increase in expenses required to improve the comfort of the child.^26–28^ Furthermore, the caregiving burden often does not reduce as the child grows and can also impact the health of main carers.^26 29–34^ With this in mind and had it been possible to extend the perspective of this analysis from one of the health service to wider society, the findings reported here would likely have been strengthened.

Second, these analyses used a subsample (130/184, 71%) of UK data from the 184 parents who consented to participate in the 6–7 years TOBY Children follow-up study. Analyses showed no significant differences between children whose parents completed the questionnaire in the UK, and in non-UK countries. There were also no differences between the 130 UK children used in the analysis, and the 39 children whose parents consented but did not return the questionnaire. Furthermore, and when looking between trial arms at differences in clinical outcomes for children in responding and non-responding families, the direction and magnitude of the differences observed were not dissimilar. Had these 39 children been included in the analysis, one might hypothesise that the findings would not have altered substantially.

Third, it is important to consider the implications for the cost results, had it been possible to include the 45/229 (20%) surviving TOBY Children lost to follow-up. These children had more severe abnormalities on EEG at trial entry and lower scores on the Mental Development Index at 18 months.^5^ Based on the results in table 4 which showed higher costs accompany higher levels of disability, one can hypothesise that the mean cost estimates reported here would likely be greater had data from these children been available. With two-fifths of the 45 children in the hypothermia arm, and three-fifths in the control arm, it is also likely that the magnitude of the cost difference favouring hypothermia would increase.

Fourth, the study relied on parents recalling healthcare contacts over the previous 6 months. We acknowledge that data for the analysis could have been obtained from routine national secondary sources; however, this approach had both cost and time implications and would also probably not have yielded the same richness of data provided by parents on additional care inputs required by their children (eg, respite carers, educational psychologists). Finally, some resource use data were missing and imputation of these data had a noticeable impact on a small number of cost categories. However, the magnitude and direction of changes were intuitive given the nature of the missing data and the observed distributions of complete data which informed the imputed values.

## Conclusions

This study is the first to report primary, community and secondary healthcare costs in children surviving 6–7 years after randomisation to hypothermia or standard care alone for perinatal asphyxia. Hypothermia was associated with lower costs, although the reduction did not achieve statistical significance and was sensitive to outlying observations. The study has also been able to quantify how healthcare costs increase with greater levels of disability and we believe this information will be both important and useful to those involved in planning patient care. In conclusion, this work provides previously unavailable data of interest to clinicians, health policy makers and health economists who may now wish to re-evaluate the long-term economic consequences of hypothermia for perinatal asphyxia.
