# Supplementary material for: Hypothermia for perinatal asphyxia: trial-based resource use and costs at 6–7 years
Source: Arch Dis Child Fetal Neonatal Ed. 2018 Jul 11;104(3):F285–92. doi: 10.1136/archdischild-2017-314685 (PMC6764253; doi:10.1136/archdischild-2017-314685)
Supplement: Supplementary file 1 [file fetalneonatal-2017-314685supp001.docx]

**Online Web Appendix**

**Missing Data and Multiple Imputation**

Each healthcare contact count variable had an associated indicator variable showing whether a child had any contact with that professional / service. Consequently, two types of missingness were noted; for some children both count and indicator variables were missing whereas for others, a yes was recorded for a contact but the number of contacts was missing.

Multiple imputation utilises regression-based approaches to predict a number of different values for each missing data point, in essence creating several different datasets.^1 2^ The approach retains the correlation structure between variables and by imputing numerous values explicitly accounts for uncertainty not only within the prediction model parameters (i.e. that they are derived only from a sample of data) but also for stochastic variability (i.e. that patients with the same values of covariates within a prediction model may not necessarily have the same outcome).

Here, MI was conducted simultaneously for the health-related quality of life HUI utility data also collected using the questionnaire (reported elsewhere), and for the resource use data. Fifty values were imputed for each missing data point by specifying separate regression models for each variable with missing data. Within each model, the remaining variables with missing data (HUI utility and healthcare contact variables) were used as predictors along with variables having complete data upon trial entry (e.g. gender, birthweight, head circumference) and study clinical endpoints at 6-7 years (e.g. disability level and IQ ≥ 85). For healthcare contacts, a two-stage imputation approach was required to appropriately handle the two types of missingness noted above. Firstly, and for each contact type, imputation was performed for all cases where data were missing data on the number of contacts. This first stage of imputation drew values from all corresponding patients with complete data for the same contact. It was possible for a zero value to be imputed for a missing healthcare contact but for the associated indicator variable for that contact to indicate that at least one contact had taken place. So as not to underestimate healthcare contacts, a second stage imputation was conducted in which non-zero values were imputed for the small number of patients known to have had contacts but for whom a zero had been imputed during the first stage. Imputation was performed using prediction mean matching (using 5 nearest neighbours for part 1 and 2 nearest neighbours for part 2), and for each trial arm separately.

**References**

1. White IR, Royston P, Wood AM. Multiple imputation using chained equations: Issues and guidance for practice. *Stat Med* 2011;30(4):377-99. doi: 10.1002/sim.4067 [published Online First: 2011/01/13]

2. Faria R, Gomes M, Epstein D, et al. A guide to handling missing data in cost-effectiveness analysis conducted within randomised controlled trials. *PharmacoEconomics* 2014;32(12):1157-70. doi: 10.1007/s40273-014-0193-3

**Results**

Table A1 below shows the characteristics and 6 to 7-year clinical outcomes for three groups of children: 1) surviving children in the UK whose parents completed the follow-up questionnaire and who formed the sample for this study (n=130), 2) all surviving children whose parents completed the follow-up questionnaire (n=145), and 3) surviving children whose parents consented to the TOBY Children Study but who did not respond to or who declined the questionnaire. Also compared are parental socioeconomic characteristics at 6-7 years. No significant differences were detected between the sample used for this study (n=130) and non-UK parents returning questionnaires, or parents not responding to or declining the questionnaire.

**Table A1: Baseline demographics and selected clinical characteristics at trial entry, and parental socioeconomic characteristics at 6-7 years, for sub-samples of children with returned and missing parent questionnaires**

|  | **UK returned parent questionnaires** | **All returned parent questionnaires** | **Missing parent questionnaire** |
| --- | --- | --- | --- |
|  | **n=130** | **n=145** | **n=39** |
| *Trial allocation* |  |  |  |
| Control Group | 63 (48) | 70 (48) | 16 (41)* |
| Hypothermia Group | 67 (52) | 75 (52) | 23 (59)* |
| **Baseline demographics and characteristics at trial entry** | | | |
| *Male sex, n (%):* | 76 (58) | 88 (61) | 25 (64) |
| Missing | 0 | 0 | 0 |
| *Gestational age (weeks):* |  |  |  |
| Median (IQR) | 40.1 (39.1-41.1) | 40.1 (39.1-41.1) | 40.6 (39-41.3) |
| Missing | 4 | 17 | 6 |
| *Birth weight (grams):* |  |  |  |
| Median (IQR) | 3435 (3190-3860) | 3446 (3190-3828) | 3500 (3160-3950) |
| Missing | 0 | 0 | 0 |
| *Delivery complications, n (%):* | 97 (75) | 109 (76) | 25 (66) |
| Missing | 1 | 2 | 1 |
| *Apgar score ≤5 at 10 minutes, n (%):* | 77 (72) | 83 (72) | 27 (79) |
| Missing | 23 | 30 | 5 |
| **Outcomes at 6-7 years** | | | |
| *Normal neurological function, n (%):* | 77 (59) | 85 (59) | 20 (51) |
| Missing | 0 | 0 | 0 |
| *IQ ≥ 85, n (%)* | 94 (74) | 102 (72) | 25 (64) |
| Missing | 3 | 3 | 0 |
| *Overall disability†, n (%):* |  |  |  |
| None or mild | 90 (70) | 101 (71) | 26 (67) |
| Moderate or severe | 38 (30) | 42 (29) | 13 (33) |
| Missing | 2 | 2 | 0 |
| **Parental socioeconomic characteristics at 6-7 years** | | | |
| *Main carer highest qualification, n (%):* |  |  |  |
| - None of the below | 7 (5) | 8 (6) | 1 (8) |
| - Vocational qualification NVQ or CSE | 18 (14) | 18 (13) | 1 (8) |
| - O Level, GCSE or Scottish Standards | 25 (20) | 27 (19) | 5 (38) |
| - BTEC, A Levels or Scottish Highers | 12 (9) | 18 (13) | 3 (23) |
| - Diploma or HND | 17 (13) | 18 (13) | 0 (0) |
| - University degree | 27 (21) | 32 (22) | 2 (15) |
| - Postgraduate University degree | 19 (15) | 19 (13) | 1 (8) |
| - Other qualification | 3 (2) | 3 (2) | 0 (0) |
| Missing | 2 | 2 | 26 |
| *Main carer employment, n (%):* |  |  |  |
| - Employed | 63 (49) | 72 (50) | 8 (67) |
| - Self-employed | 9 (7) | 11 (8) | 2 (17) |
| - Unemployed | 24 (19) | 25 (17) | 1 (8) |
| - Other‡ | 32 (25) | 35 (24) | 1 (8) |
| Missing | 2 | 2 | 27 |
| *Main carer home, n (%):* |  |  |  |
| - Owner (mortgage) | 83 (64) | 96 (66) | 11 (85) |
| - Council rented | 18 (14) | 19 (13) | 1 (8) |
| - Private rented (furnished) | 5 (4) | 5 (3) | 0 (0) |
| - Private rented (unfurnished) | 14 (11) | 15 (10) | 1 (8) |
| - Housing society or co-operative | 4 (3) | 4 (3) | 0 (0) |
| - Other§ | 6 (5) | 6 (4) | 0 (0) |
| Missing | 0 | 0 | 26 |

IQR Inter-quartile range; IQ Intelligence Quotient; NVQ National Vocational Qualification; CSE Certificate of Secondary Education; O Level Ordinary Level; GCSE General Certificate of Secondary Education; BTEC Business and Technology Education Council; A Level Advanced Level; HND Higher National Diploma

* Amongst the 39 non-participating families, a higher proportion of children in the hypothermia group than in the control group had an IQ≥85 (16/23 (70%) v 9/16 (56%), p=0.394) and had normal neurological functioning (14/23 (61%) v 6/16 (38%), p=0.151).

†Overall disability - mild disability (an IQ score of 70 to 84, level 1 gross motor function [is able to walk independently but may have some gait abnormalities], or abnormality in one or both eyes with normal

or nearly normal vision); moderate disability - (an IQ score of 55 to 69, level 2 or 3 gross motor function [has minimal ability to perform gross motor skills or requires assistance with walking], or moderately reduced vision); severe disability (an IQ score of <55, level 4 or 5 gross motor function [needs adaptive seating or has severely limited mobility], or no useful vision)

‡Open ended question (responses included housewife, carer, etc.)

§Open ended question

To help provide an understanding of the implications of the multiple imputation, table A2 summarises healthcare contacts and associated costs estimated using only complete UK data for each variable. A comparison of these estimates with those obtained using imputation and shown in Table 3 of the main paper reveals that following imputation, the magnitude and / or direction of the mean cost differences for some variables is altered. This occurred as a result of a number of factors including the nature of the missingness. For some healthcare services, data on the number of contacts was missing but additional information was available to indicate that at least one contact had taken place (see above). For these cases non-zero values were imputed using complete data from other patients with the same type of contact. When the proportion of patients with this type of missingess was imbalanced between the two trial arms, the potential existed for the number of visits in one arm to be altered substantially from that observed when only complete data were considered. In turn, the associated cost estimates and the cost differential between the two arms also changed.

To illustrate, when using only complete cases, the mean cost difference (95% CI) for community nurse visits was -£35 (-£144 to £73) in favour of the hypothermia group (Table A2). Following imputation however, the mean number of visits in the hypothermia arm almost doubled from 0.44 to 0.85 and this changed the mean cost difference to £6 (-£130 to £141) in favour of the control group (Table 3, main paper).

This change occurred because of the 10 children with missing data in the hypothermia group, four were known to have had contact with a community nurse (but the number of contacts was not recorded); 10 children were also missing data in the control group but it was not known whether any had had contact with a community nurse (see Table A3). In the hypothermia arm therefore this effectively doubled the number of children with known contacts from four to eight (see Table A4). After imputing visit numbers for these additional children using complete data on visit numbers from children who saw a community nurse (Table A4 shows the mean and (SD) visit numbers for these children to be 6.25 (9.18)), the overall mean number of visits, along with the cost in the hypothermia arm, increased.

Similarly the mean (SD) number of SALT visits in the hypothermia arm more than doubled from 1.40 (7.02) to 3.03 (1.47) following imputation; the mean cost difference changed from -£77 (-£302 to £148) in favour of hypothermia (Table A2) to £37 (-£279 to £353) in favour of the control group (Table 3, main paper). In this instance, the number of children who were known to have seen a SALT specialist but who were without recorded visit numbers was similar in both arms (8 v 7, see Table A3). The data informing the distributions used to impute visit numbers for these children in each trial arm however were different. Table A4 shows that for the 20 children with complete data who were seen by a SALT specialist in the control arm, the mean (SD) number of contacts was 5.55 (6.53). In contrast, the corresponding mean (SD) number of visits for the 8 children in the hypothermia arm with SALT contacts was much higher at 9.13 (16.68) on account of one parent reporting 50 contacts (an average of almost 2 visits per week) over the six-month recall period. The impact of this was that imputed values for children in the hypothermia arm with known SALT contacts but unreported visit numbers, were much greater than those in the control arm.

For a small number of other variables including Hospital Day Unit and hospital outpatient clinic attendances, costs in the hypothermia arm similarly increased following imputation. Hospital inpatient admissions, which, as shown in the main paper drive most of the total cost difference between the two arms of the trial, were complete for all but two children in the hypothermia arm (for both data on whether any admissions had taken place were missing). Imputation therefore had little or no impact upon these results as can be seen by comparing the data in Table 3 of the main paper and Table A2 below.

**Table A2: Analysis of health-care resource use and costs in the hypothermia and control groups (UK data only n=130) using only cases with complete data**

| **Resource use category** | **Control Group**  **(n=63)** | | **Hypothermia Group (n=67)** | |  | **Control Group (n=63)** | **Hypothermia Group (n=67)** |  |
| --- | --- | --- | --- | --- | --- | --- | --- | --- |
|  | **Complete,**  **n (%)** | **Mean (SD)** | **Complete,**  **n (%)** | **Mean (SD)** | **Mean difference**  **(95% CIs)** | **Mean Cost (SD)** | **Mean Cost (SD)** | **Mean cost difference (95% CIs)** |
| **Primary care:** |  |  |  |  |  |  |  |  |
| GP visits | 57 | 1.44 (1.90) | 64 | 1.34 (2.07) | -0.09 (-0.81 to 0.62) | £52 (£68) | £48 (£75) | -£3 (-£29 to £22) |
| Practice nurse visits | 52 | 0.10 (0.30) | 58 | 0.26 (0.89) | 0.16 (-0.09 to 0.42) | £1 (£3) | £3 (£10) | £2 (-£1 to £5) |
| **Community care:** |  |  |  |  |  |  |  |  |
| Health visitor visits | 52 | 0.00 (0.00) | 57 | 0.14 (0.61) | 0.14 (-0.03 to 0.31) | £0 (£0) | £8 (£36) | £8 (-£2 to £18) |
| Community nurse visits | 53 | 0.75 (2.47) | 57 | 0.44 (2.67) | -0.32 (-1.29 to 0.66) | £84 (£276) | £49 (£297) | -£35 (-£144 to £73) |
| Community paed. visits | 55 | 0.29 (0.74) | 59 | 0.49 (1.59) | 0.20 (-0.27 to 0.67) | £81 (£205) | £136 (£441) | £56 (-£74 to £185) |
| Optician visits | 53 | 0.26 (0.49) | 56 | 0.41 (0.93) | 0.15 (-0.14 to 0.43) | £6 (£10) | £9 (£20) | £3 (-£3 to £9) |
| Orthoptist visits | 52 | 0.31 (0.78) | 55 | 0.20 (0.76) | -0.11 (-0.40 to 0.19) | £18 (£45) | £12 (£44) | -£6 (-£23 to £11) |
| Physiotherapist visits | 49 | 2.18 (5.38) | 57 | 1.67 (6.95) | -0.52 (-2.94 to 1.91) | £191 (£469) | £145 (£607) | -£45 (-£257 to £166) |
| SALT visits | 50 | 2.22 (4.91) | 52 | 1.40 (7.02) | -0.82 (-3.20 to 1.57) | £210 (£463) | £133 (£662) | -£77 (-£302 to £148) |
| **Secondary care:** |  |  |  |  |  |  |  |  |
| A&E visits | 58 | 0.34 (1.07) | 56 | 0.14 (0.40) | -0.20 (-0.50 to 0.10) | £64 (£200) | £27 (£75) | -£38 (-£94 to £19) |
| Hospital Day Unit visits | 59 | 0.10 (0.55) | 52 | 0.08 (0.33) | -0.02 (-0.20 to 0.15) | £76 (£410) | £58 (£250) | -£19 (-£148 to £111) |
| Hospital outpatient visits | 55 | 0.56 (1.48) | 50 | 0.44 (1.23) | -0.12 (-0.65 to 0.41) | £110 (£287) | £86 (£239) | -£24 (-£127 to £79) |
| Hospital in-patient days | 63 | 2.21 (9.95) | 65 | 0.71 (3.13) | -1.50 (-4.06 to 1.07) | £1116 (£5306) | £305 (£1349) | -£811 (-£2156 to £534) |
| **Misc. clinics/therapist sessions*****:** |  |  |  |  |  |  |  |  |
| Occupational therapist visits | 60 | 0.62 (2.71) | 66 | 0.39 (1.64) | -0.22 (-1.00 to 0.56) | £81 (£355) | £52 (£215) | -£29 (-£132 to £73) |
| Parent support worker/Special educational needs worker | 62 | 0.73 (5.09) | 65 | 0.12 (0.78) | -0.61 (-1.87 to 0.66) | £38 (£265) | £6 (£41) | -£31 (-£97 to £34) |
| Dietician visits | 63 | 0.05 (0.38) | 67 | 0.10 (0.55) | 0.06 (-0.11 to 0.22) | £4 (£31) | £8 (£45) | £5 (-£9 to £18) |
| Dentist visits | 63 | 0.02 (0.13) | 67 | 0.04 (0.21) | 0.03 (-0.03 to 0.09) | £1 (£7) | £2 (£11) | £2 (-£2 to £5) |
| Educational psychologist visits | 63 | 0.02 (0.13) | 67 | 0.01 (0.12) | 0.00 (-0.04 to 0.04) | £1 (£11) | £1 (£10) | £0 (-£4 to £4) |
| Home Respite Team visits | 63 | 0.38 (3.02) | 67 | 0.00 (0.00) | -0.38 (-1.11 to 0.35) | £73 (£581) | £0 (£0) | -£73 (-£213 to £67) |
| Social Worker visits | 63 | 0.00 (0.00) | 67 | 0.09 (0.73) | 0.09 (-0.09 to 0.27) | £0 (£0) | £7 (£58) | £7 (-£7 to £22) |
| Support for physical disability visits | 63 | 0.32 (2.52) | 67 | 0.00 (0.00) | -0.32 (-0.93 to 0.29) | £61 (£484) | £0 (£0) | -£61 (-£178 to £56) |

GP: General Practice; paed: paediatrics; SALT: Speech and language therapy; A&E: Accident and Emergency; CIs: parametric confidence intervals; *Miscellaneous clinics/therapist sessions are from a section of the questionnaire, which was optional to complete. We assume completeness of data for this section.

**Table A3 Breakdown of amount and type of missing healthcare contact data**

|  | **Control Group (n=63)** | | | **Hypothermia Group (n=67)** | | |
| --- | --- | --- | --- | --- | --- | --- |
|  | **Missing, n** | **Of the missing….** | | **Missing, n** | **Of the missing….** | |
|  |  | **Unknown if contact occurred**  **n (%)** | **Contact known to have occurred but number not recorded**  **n (%)** |  | **Unknown if contact occurred**  **n (%)** | **Contact known to have occurred but number not recorded**  **n (%)** |
| **Primary care:** |  |  |  |  |  |  |
| GP visits | 6 (9.5%) | 2 (33.3%) | 4 (66.7%) | 3 (4.5%) | 1 (33.3%) | 2 (66.7%) |
| Practice nurse visits | 11 (17.5%) | 10 (90.9%) | 1 (9.1%) | 9 (13.4%) | 9 (100.0%) | 0 (0.0%) |
| **Community care:** |  |  |  |  |  |  |
| Health visitor visits | 11 (17.5%) | 10 (90.9%) | 1 (9.1%) | 10 (14.9%) | 8 (80.0%) | 2 (20.0%) |
| Community nurse visits | 10 (15.9%) | 10 (100.0%) | 0 (0.0%) | 10 (14.9%) | 6 (60.0%) | 4 (40.0%) |
| Community paed. visits | 8 (12.7%) | 7 (87.5%) | 1 (12.5%) | 8 (11.9%) | 5 (62.5%) | 3 (37.5%) |
| Optician visits | 10 (15.9%) | 10 (100.0%) | 0 (0.0%) | 11 (16.4%) | 10 (90.9%) | 1 (9.1%) |
| Orthoptist visits | 11 (17.5%) | 11 (100.0%) | 0 (0.0%) | 12 (17.9%) | 11 (91.7%) | 1 (8.3%) |
| Physiotherapist visits | 14 (22.2%) | 6 (42.9%) | 8 (57.1%) | 10 (14.9%) | 6 (60.0%) | 4 (40.0%) |
| SALT visits | 13 (20.6%) | 5 (38.5%) | 8 (61.5%) | 15 (22.4%) | 8 (53.3%) | 7 (46.7%) |
| **Secondary care:** |  |  |  |  |  |  |
| A&E visits | 5 (7.9%) | 3 (60.0%) | 2 (40.0%) | 11 (16.4%) | 10 (90.9%) | (9.1%) |
| Hospital Day Unit visits | 4 (6.3%) | 4 (100.0%) | 0 (0.0%) | 15 (22.4%) | 14 (93.3%) | 1 (6.7%) |
| Hospital outpatient visits | 8 (12.7%) | 2 (25.0%) | 6 (75.0%) | 17 (25.4%) | 9 (52.9%) | 8 (47.1%) |
| Hospital in-patient days | 0 (0.0%) | 0 (0.0%) | 0 (0.0%) | 2 (3.0%) | 2 (100.0%) | 0 (0.0%) |
| **Misc. clinics/therapist sessions*****:** |  |  |  |  |  |  |
| Occupational therapist visits | 3 (4.8%)† | 0 (0.0%) | 3 (100.0%) | 1 (1.5%)† | 0 (0.0%) | 1 (100.0%) |
| Parent support worker/Special educational needs worker | 1 (1.6%)† | 0 (0.0%) | 1 (100.0%) | 2 (3.0%)† | 0 (0.0%) | 2 (100.0%) |
| Dietician visits | 0 (0.0%) | 0 (0.0%) | 0 (0.0%) | 0 (0.0%) | 0 (0.0%) | 0 (0.0%) |
| Dentist visits | 0 (0.0%) | 0 (0.0%) | 0 (0.0%) | 0 (0.0%) | 0 (0.0%) | 0 (0.0%) |
| Educational psychologist visits | 0 (0.0%) | 0 (0.0%) | 0 (0.0%) | 0 (0.0%) | 0 (0.0%) | 0 (0.0%) |
| Home Respite Team visits | 0 (0.0%) | 0 (0.0%) | 0 (0.0%) | 0 (0.0%) | 0 (0.0%) | 0 (0.0%) |
| Social Worker visits | 0 (0.0%) | 0 (0.0%) | 0 (0.0%) | 0 (0.0%) | 0 (0.0%) | 0 (0.0%) |
| Support for physical disability visits | 0 (0.0%) | 0 (0.0%) | 0 (0.0%) | 0 (0.0%) | 0 (0.0%) | 0 (0.0%) |

GP: General Practice; paed: paediatrics; SALT: Speech and language therapy; A&E: Accident and Emergency; *Miscellaneous clinics/therapist sessions are from a section of the questionnaire, which was optional to complete. We assume completeness of data for this section. †Contact known to have taken place but the number of contacts is missing.

**Table A4: Breakdown of health-care resource use over the last 6 months in the hypothermia and control groups for survivors at 6-7 years (UK data only n=130) for only those children who consumed the category of resource use**

| **Resource use category** | **Control Group (n=63)** | | | | **Hypothermia Group (n=67)** | | | | **Mean difference**  **(95% CI)** |
| --- | --- | --- | --- | --- | --- | --- | --- | --- | --- |
|  | **n** | **Min.** | **Max.** | **Mean (SD)** | **n** | **Min.** | **Max.** | **Mean (SD)** |  |
| **Primary care:** |  |  |  |  |  |  |  |  |  |
| GP visits | 35 | 1 | 12 | 2.34 (1.94) | 36 | 1 | 10 | 2.39 (2.27) | 0.05 (-0.96 to 1.05) |
| Practice nurse visits | 5 | 1 | 1 | 1.00 (0.00) | 8 | 1 | 6 | 1.88 (1.73) | 0.88 (-0.85 to 2.60) |
| **Community care:** |  |  |  |  |  |  |  |  |  |
| Health visitor visits | - | - | - | - | 4 | 1 | 4 | 2.00 (1.41) | 2.00 (-) |
| Community nurse visits | 8 | 1 | 15 | 5.00 (4.60) | 4 | 1 | 20 | 6.25 (9.18) | 1.25 (-7.39 to 9.89) |
| Community paed. visits | 10 | 1 | 4 | 1.60 (0.97) | 12 | 1 | 10 | 2.42 (2.87) | 0.82 (-1.17 to 2.81) |
| Optician visits | 13 | 1 | 2 | 1.08 (0.28) | 16 | 1 | 6 | 1.44 (1.26) | 0.36 (-0.37 to 1.10) |
| Orthoptist visits | 10 | 1 | 4 | 1.60 (1.07) | 6 | 1 | 5 | 1.83 (1.60) | 0.23 (-1.19 to 1.66) |
| Physiotherapist visits | 16 | 1 | 25 | 6.69 (7.77) | 9 | 2 | 50 | 10.56 (15.26) | 3.87 (-5.59 to 13.32) |
| SALT visits | 20 | 1 | 25 | 5.55 (6.53) | 8 | 1 | 50 | 9.13 (16.68) | 3.58 (-5.28 to 12.43) |
| **Secondary care:** |  |  |  |  |  |  |  |  |  |
| A&E visits | 10 | 1 | 7 | 2.00 (1.89) | 7 | 1 | 2 | 1.14 (0.38) | -0.86 (-2.41 to 0.70) |
| Hospital Day Unit visits | 3 | 1 | 4 | 2.00 (1.73) | 3 | 1 | 2 | 1.33 (0.58) | -0.67 (-3.59 to 2.26) |
| Hospital outpatient visits | 10 | 1 | 7 | 3.10 (2.08) | 8 | 1 | 6 | 2.75 (1.83) | -0.35 (-2.34 to 1.64) |
| Hospital in-patient days | 7 | 2 | 58 | 19.86 (24.78) | 7 | 1 | 20 | 6.57 (7.72) | -13.29 (-34.66 to 8.09) |
| **Misc. clinics/therapist sessions*****:** |  |  |  |  |  |  |  |  |  |
| Occupational therapist visits | 7 | 2 | 20 | 5.29 (6.58) | 6 | 2 | 12 | 4.33 (3.83) | -0.95 (-7.69 to 5.78) |
| Parent support worker/Special educational needs worker | 3 | 2 | 40 | 15 (21.66) | 2 | 2 | 6 | 4.00 (2.83) | -11.00 (-62.59 to 40.59) |
| Dietician visits | 1 | 3 | 3 | 3 (0.00) | 3 | 1 | 4 | 2.33 (1.53) | -0.67 (-8.26 to 6.92) |
| Dentist visits | 1 | 1 | 1 | 1 (0.00) | 3 | 1 | 1 | 1 (0.00) | 0 (-) |
| Educational psychologist visits | 1 | 1 | 1 | 1 (0.00) | 1 | 1 | 1 | 1 (0.00) | 0 (-) |
| Home Respite Team visits† | 1 | 24 | 24 | 24 (0.00) | - | - | - | - | -24.00 (-) |
| Social Worker visits‡ | - | - | - | - | 1 | 6 | 6 | 6 (0.00) | 6.00 (-) |
| Support for physical disability visits | 1 | 20 | 20 | 20 (0.00) | - | - | - | - | -20.00 (-) |

GP: General Practice; paed: paediatrics; SALT: Speech and language therapy; A&E: Accident and Emergency; CI: parametric confidence intervals

*Miscellaneous clinics/therapist sessions are from a section of the questionnaire, which was optional to complete. We assume completeness of data for this section.

†Reported by one patient in the control group but the number of contacts was missing. One eight hour day of respite care per week was assumed.

‡Reported by one patient in the hypothermia group but the number of contacts was missing. One hourly visit per month was assumed.

**Modelling the relationship between costs and disability levels**

Total healthcare costs can be calculated using the coefficients from the general linear regression model by summing the products of each coefficient and corresponding covariate value and then exponentiating the resulting figure. In the following example, costs were modelled for a male, born at 40 weeks, having delivery complications, a birth weight of 3400 grams, receiving hypothermia and having a mild disability. From Table 4 of the main paper, the coefficient values in the model for the constant, trial arm, delivery complications, gestational age, birth weight, gender, and mild disability, are respectively as follows: 11.84964, 0.5520007, 0.5974293, -0.1449353, -0. 0002374, -0. 285753, 0. 8407097. By including the covariate values, total healthcare costs are calculated using the formula below:

Total healthcare costs = exp(11.84964 + (0.5520007 x 1) + (0.5974293 x 1) + (-0.1449353*40) + (-0. 0002374*3400) + (-0.285753 x 1) + (0.8407097 x 1)) = £1043
